# From Conventional to Smart Prosthetics: Redefining Complete Denture Therapy Through Technology and Regenerative Science

**DOI:** 10.3390/medicina61061104

**Published:** 2025-06-18

**Authors:** Andrea Bors, Simona Mucenic, Adriana Monea, Alina Ormenisan, Gabriela Beresescu

**Affiliations:** Faculty of Dental Medicine, George Emil Palade University of Medicine, Pharmacy, Science, and Technology of Targu Mures, 38 Gh. Marinescu Str., 540139 Târgu Mureș, Romania; andrea.bors@umfst.ro (A.B.); simona.mucenic@umfst.ro (S.M.); adriana.monea@umfst.ro (A.M.); alina.ormenisan@umfst.ro (A.O.)

**Keywords:** complete denture, digital dentistry, regenerative dentistry, smart prosthetics, CAD–CAM, edentulism rehabilitation

## Abstract

*Background and Objectives:* Complete dentures remain a primary solution for oral rehabilitation in aging and medically compromised populations. The integration of digital workflows, regenerative materials, and smart technologies is propelling prosthodontics towards a new era, transcending the limitations of traditional static prostheses. *Materials and Methods:* This narrative review synthesizes historical developments, current practices, and future innovations in complete denture therapy. A comprehensive review of literature from PubMed, Scopus, and Web of Science (2000–2025) was conducted, with a focus on materials science, digital design, patient-centered care, artificial intelligence (AI), and sustainable fabrication. *Results:* Innovations in the field include high-performance polymers, CAD–CAM systems, digital impressions, smart sensors, and bioactive liners. Recent trends in the field include the development of self-monitoring prostheses, artificial intelligence (AI)-driven design platforms, and bioprinted regenerative bases. These advances have been shown to enhance customization, durability, hygiene, and patient satisfaction. However, challenges persist in terms of accessibility, clinician training, regulatory validation, and ethical integration of digital data. *Conclusions:* The field of complete denture therapy is undergoing a transition toward a new paradigm of prosthetics that are personalized, intelligent, and sustainable. To ensure the integration of these technologies into standard care, ongoing interdisciplinary research, clinical validation, and equitable implementation are imperative.

## 1. Introduction

Complete dentures persist as a basic solution for oral rehabilitation in edentulous patients, particularly among elderly individuals or those with systemic, financial, or anatomical limitations that preclude implant therapy. Despite the increasing availability of implant-supported prostheses, full dentures remain widely used due to their accessibility, versatility, and cost-effectiveness [1,2].

A review of global demographic trends reveals a persistent high prevalence of edentulism. According to the World Health Organization (WHO), approximately 30% of individuals over the age of 65 in many industrialized nations are completely edentulous. Edentulism has been associated with several adverse consequences, including reduced masticatory function, nutritional deficiencies, diminished self-esteem, and impaired quality of life [3]. Tooth loss is associated with nutritional deficiencies, decreased self-esteem, and impaired social interaction, highlighting the importance of functional and aesthetic prosthetic rehabilitation [4]. Smart prosthetics were developed in the context of complete dentures as prosthetic devices that incorporate digital design, advanced materials, and/or embedded microsensors capable of acquiring data on oral conditions (e.g., occlusal load, pH, temperature) and wirelessly transmitting this information to support personalized care [5,6].

While previous reviews have examined specific components, such as digital dentures or implant-supported overdentures, there has been a paucity of reviews that comprehensively investigate the integration of emerging technologies and patient-centered care within the broader context of full removable prostheses. Furthermore, the mounting interest in sustainability and data ethics in dentistry underscores the need for an updated synthesis.

The objective of this study is to identify and address a significant research gap by conducting an integrated analysis of traditional and emerging paradigms in complete denture therapy. It offers a comprehensive evaluation of the historical underpinnings of this therapy, contemporary best practices, and future trends in the field. This analysis encompasses the integration of smart technologies, the application of artificial intelligence in workflow optimization, the utilization of biomimetic materials, and the exploration of sustainable fabrication methods. By doing so, this review aims to inform clinicians, researchers, and policymakers about the challenges and opportunities shaping the future of edentulous rehabilitation.

## 2. Material and Methods

The present narrative review was conducted for the purpose of exploring changes, current practices, and emerging directions in complete denture therapy. The aim of the review was to synthesize data on materials, fabrication techniques, patient-centered innovations, and the integration of digital and regenerative technologies in prosthodontics.

### 2.1. Literature Search Strategy

A structured literature search was conducted using PubMed, Scopus, and Web of Science for studies published between 2000 and February 2025. A comprehensive list of contemporary subjects in the field of prosthodontics would include, but is not limited to, the following keywords, which were used for the search: complete dentures, digital dentures, CAD–CAM, regenerative prosthodontics, smart prosthetics, biomimetic dentures, artificial intelligence in prosthodontics, and sustainable dental materials. Boolean operators (AND/OR) were employed to refine the results obtained.

### 2.2. Inclusion and Exclusion Criteria

The criteria established to determine inclusion and exclusion are described below.

The inclusion criteria for this study are as follows: peer-reviewed articles published in English; studies addressing complete dentures in relation to materials, digital workflows, smart technology, patient experience, sustainability, or regenerative strategies; review articles, experimental studies, clinical trials, and relevant case reports.

The following criteria were used to determine exclusion from the study: studies focused solely on partial or fixed prosthodontics; non-English-language publications; abstracts without accessible full text.

### 2.3. Data Extraction and Synthesis

Articles were selected based on title and abstract screening, which was followed by full-text evaluation. The following key themes were extracted: material advancements, fabrication technologies, patient-centered care models, digital innovations, AI applications, and sustainable practices. A narrative synthesis was conducted to integrate historical context, current applications, and prospective advancements.

### 2.4. Limitations

As a narrative review, this study does not include a formal risk of bias assessment or meta-analysis. However, the integration of multiple study types and thematic comparison enhances its relevance for clinical, academic, and innovation-focused audiences.

## 3. Discussions

### 3.1. Brief History of Complete Dentures

The development of complete dentures spans centuries, reflecting both the evolution of dental materials and the general advancement of prosthodontic science. The earliest records of artificial teeth date back to ancient civilizations: the Etruscans in the 7th century BC made dentures from animal teeth or human teeth bound with gold wire [7]. However, true complete dentures, which replaced all the teeth in an arch, did not appear much later.

In the 18th century, ivory (often from hippopotamus tusks) was carved into denture bases and fitted with human or animal teeth. These dentures, though rudimentary, laid the foundation for modern concepts of full-arch replacement. One of the most famous early wearers of dentures was George Washington, whose sets reportedly included materials such as ivory, gold, and even lead [7].

The 19th century saw significant advances with the introduction of vulcanized rubber in 1839 by Charles Goodyear. Vulcanite provided a malleable, inexpensive material for denture bases, replacing expensive metals and allowing wider access to prosthetic care [8].

A major milestone came in the 1930s with the development of polymethyl methacrylate (PMMA), a durable, aesthetic, and biocompatible acrylic resin that quickly became the standard denture-base material. PMMA revolutionized denture fabrication, offering advantages in cost, ease of fabrication, and comfort [9].

In the late 20th and early 21st centuries, digital technologies were introduced, allowing for more precise, reproducible, and aesthetically pleasing dentures. This digital evolution marked a transition from artisanal to data-driven prosthodontics, improving both efficiency and patient outcomes [10].

Thus, from ancient ivory frameworks to AI-driven digital prostheses, complete dentures have evolved in parallel with materials science, engineering, and clinical innovations.

### 3.2. Current State-of-the-Art

#### 3.2.1. Materials and Fabrication

Conventional Dentures. Conventional complete dentures are most often manufactured using heat-polymerized polymethyl methacrylate (PMMA) for the denture base; this remains the material of choice due to its favorable aesthetics, ease of fabrication, and acceptable mechanical strength. Despite its long-standing clinical use, PMMA has known drawbacks, including polymerization shrinkage, low impact strength, and susceptibility to microbial colonization, particularly by *Candida albicans* [11,12].

Artificial teeth used in conventional dentures are typically made of acrylic resin or porcelain. Acrylic resin teeth bond well to PMMA bases and offer good wear resistance, while porcelain teeth are harder, more aesthetic, and more wear-resistant, but pose challenges due to poor bonding and increased brittleness [13].

Advanced Materials. Recent advances have focused on improving the mechanical and biological performance of denture materials.

- High-impact PMMA resins incorporate rubber or other toughening agents to improve fracture resistance without significantly altering processing protocols [14].
- Glass fiber, carbon fiber, and nanofiller-reinforced PMMAs have shown promise in improving flexural strength and fatigue resistance while maintaining biocompatibility [15,16].
- Flexible denture bases such as nylon-based polyamides (e.g., Valplast^®^) offer superior comfort and elasticity, especially for patients with undercuts or soft-tissue sensitivity. However, they can compromise long-term dimensional stability and are more difficult to adjust and reline [17].
- Antimicrobially modified resins incorporating silver nanoparticles, chlorhexidine, or quaternary ammonium compounds are being investigated for their ability to reduce biofilm formation and maintain long-term oral hygiene [18].

These innovations aim to address clinical issues such as midline fractures, microbial colonization, and patient discomfort while expanding the range of aesthetic and functional options.

Digital dentures. The integration of computer-aided design and computer-aided manufacturing (CAD–CAM) technologies into the fabrication of full denture prosthetics represents a significant evolution from traditional analogue methods. Historically, full denture fabrication involved multiple clinical and laboratory steps that were time-consuming and prone to dimensional inaccuracies. In contrast, CAD–CAM workflows offer increased precision, reproducibility, and efficiency, making them a cornerstone of modern prosthodontics [10].

CAD–CAM systems use digital records, including intraoral or extraoral scans, to design and fabricate dental restorations using either subtractive (milling) or additive (3D printing) methods. Milled dentures, fabricated from prepolymerized PMMA blocks, are particularly valued for their superior fit, improved material properties, and reduced porosity due to the elimination of polymerization shrinkage. Studies have shown that milled dentures exhibit greater mechanical strength and lower residual monomer content, contributing to improved patient safety and long-term function [10,19,20,21,22].

Beyond fabrication, CAD software (Model Builder, Dental Systems, 3Shape) enables virtual articulation, digital tooth arrangement, and simulation of occlusal schemes, improving the accuracy of prosthesis design. Digital workflows also reduce the number of patient visits, making them particularly suitable for the elderly or medically compromised. In addition, archived datasets allow for easy reproduction of prostheses without the need to take re-impressions, which greatly benefits institutionalized or dependent patients [6].

Despite its benefits, CAD–CAM adoption faces challenges, including high initial costs, limited options for aesthetic customization, and a learning curve for clinicians and technicians. Nevertheless, as materials science and software platforms continue to evolve, CAD–CAM full dentures are expected to become the standard of care in edentulous rehabilitation.

Implant-supported overdentures are now considered the gold standard for mandibular edentulism in many cases. As recommended by the McGill Consensus Statement [23], a two-implant overdenture significantly improves retention, stability, masticatory efficiency, and patient-reported quality of life compared to conventional mandibular dentures.

Implant-retained dentures reduce bone resorption, enhance proprioception, and allow for reduced denture volume, which improves comfort and speech. However, they are contraindicated in some patients due to systemic disease, inadequate bone, or financial concerns. Surgical and prosthetic complications such as attachment wear and implant maintenance should be considered [24].

Immediate Dentures. Immediate dentures are prostheses placed immediately after tooth extraction that provide aesthetic and functional benefits by preventing a period of edentulism. They help maintain facial appearance, protect extraction sites, and facilitate early masticatory function. Immediate dentures are often preferred for psychological and social reasons, particularly in the case of anterior tooth loss [25].

However, the unpredictable nature of post-extraction bone resorption and tissue remodeling requires frequent relines and possible remakes [26]. The success of immediate dentures depends on accurate diagnosis, proper patient counselling, and close follow-up during the healing period.

#### 3.2.2. Patient-Centered Care

The philosophy of prosthetic care has evolved significantly in recent decades. Beyond the traditional focus on replacing missing teeth, modern prosthetics emphasizes patient-centered care—an approach that integrates technical excellence with the individual needs, experiences, and expectations of the patient. Within this framework, prosthesis-centered care emerges as a model that prioritizes functionality, comfort, aesthetics, psychological well-being, and overall quality of life for the patient during prosthetic rehabilitation.

Restoring essential oral functions remains a fundamental goal. Properly fabricated dentures should improve chewing, speaking, and swallowing. Studies have shown that improving the fit of dentures and occlusal balance has a direct impact on chewing efficiency, food intake, and overall health [27].

Beyond functionality, aesthetic and psychosocial factors are essential to the success of prosthetic treatment. Dentures influence facial support and prevent the “sunken” facial appearance often associated with edentulism [28]. Individualized tooth selection, color matching, and natural gingival characterization can enhance psychological acceptance and improve self-esteem. Dentures are not just a replacement for missing teeth; they help restore the patient’s personal identity.

Psychological acceptance of a denture is an essential factor in the success of treatment. Many patients experience fear, anxiety, or uncertainty about wearing dentures. Counselling for adaptation—setting realistic expectations, providing encouragement, and offering a gradual wearing schedule—can significantly reduce rates of denture abandonment [29]. Empathetic communication and active listening to the patient’s concerns promote trust, adherence, and satisfaction.

In clinical practice, denture-centered care begins with a comprehensive assessment of the patient. The use of validated patient-reported outcome measures (PROMs), such as the Oral Health Impact Profile for Edentulous Patients (OHIP-EDENT), quantifies the impact of oral diseases on patients’ quality of life [30].

The benefits of a denture-centered care model are significant. Patients report higher satisfaction, better acceptance of dentures, and improved oral-health-related quality of life (OHRQoL). Clinicians also experience fewer complications, stronger doctor–patient relationships, and better long-term outcomes.

Looking ahead, digital innovations promise to further improve denture-centered care. CAD–CAM technologies and artificial intelligence-based workflows enable unprecedented customization of dentures to meet individual anatomical and aesthetic needs. Teledentistry could soon facilitate remote monitoring of denture fit, while future smart dentures with built-in sensors could alert clinicians to fit issues or mucosal changes before symptoms arise.

Ultimately, denture-centered care transforms dentures into a holistic health intervention. By combining scientific precision with empathy, clinicians restore not only oral function, but also the dignity, confidence, and quality of life of their patients.

## 4. Future Perspectives

### 4.1. Biomimetic and Regenerative Dentures

The future of complete denture prosthetics is increasingly being shaped by biomimetic and regenerative innovations that seek to mimic natural tissues not only in appearance, but also in function and biology. Moving beyond traditional static prostheses, these advanced concepts aim to develop dynamic, tissue-compatible systems that promote health, comfort, and integration with the oral environment. The convergence of materials science, tissue engineering, and bioprinting is opening new frontiers in the design of biologically interactive dentures.

A major focus is the development of biomimetic denture bases and liners that attempt to mimic the viscoelastic behavior of oral mucosa and submucosal tissues. Conventional denture bases, primarily made of polymethyl methacrylate (PMMA), are inherently rigid and can cause uneven load distribution, especially in patients with highly resorbed alveolar ridges. This can lead to mucosal irritation, compromised retention and patient discomfort. In response, there has been increased research into the development of advanced soft liners and composite bases. Silicone elastomers and polyurethane-based viscoelastic liners have been refined to exhibit damping characteristics that more closely resemble human oral tissues [31]. In addition, layered composite base materials are being explored that can simulate the mechanical gradient between mucosa, muscle, and bone, which may contribute to a more physiological load transfer and improved prosthesis adaptation [32] (Table 1).

In parallel, regenerative strategies are being investigated to improve the biological interface between the prosthesis and the underlying tissues. These regenerative tissue interfaces focus on promoting mucosal health and biointegration. Denture bases infused with therapeutic hydrogels are being developed to deliver bioactive agents such as antimicrobial peptides, anti-inflammatory compounds, or tissue regenerative molecules directly to the mucosal surface during wear. This not only combats denture-induced stomatitis and mucosal inflammation but also helps to maintain a balanced oral microbiome [33]. In addition, experimental denture-base coatings incorporating calcium phosphate, collagen, or epithelial growth factors have shown the ability to promote mucosal healing and enhance epithelial adhesion, mimicking the function of the natural basal lamina [34].

An area of innovation is the use of tissue-engineered prosthetic components. Tissue-engineering approaches have shown potential in regenerating alveolar structures using mesenchymal stem cells (MSCs) seeded on biodegradable scaffolds. These platforms can induce the formation of bone and soft tissue at the denture–mucosa interface, providing a living scaffold upon which dentures could be placed [35]. Complementing this, 3D bioprinting technologies are advancing toward the fabrication of complex, multilayered structures that mimic the periodontium or the mucosa–bone interface. These constructions may one day enable the fabrication of partially or fully integrated prostheses that biologically fuse with host tissues [36]. While these strategies remain largely experimental, they herald a paradigm shift from replacement to regeneration in prosthetic dentistry.

Another innovation inspired by the behavior of natural tissue is the development of self-healing prosthetic materials. These materials, which contain microencapsulated healing agents or possess reversible molecular cross-links, are capable of autonomously repairing minor structural defects such as microcracks or surface fissures. This technology has already shown promise in restorative dentistry and could be adapted to extend the service life of full dentures, especially in patients prone to fractures due to poor handling or parafunctional habits [37]. The application of such smart materials could reduce the frequency of prosthesis repairs, improve prosthesis longevity, and support long-term patient satisfaction.

Together, these emerging technologies are poised to redefine the boundaries of what prosthetics can achieve. Prosthodontics is rapidly evolving from work with passive devices that replace lost tissue to work with biologically integrated, self-sustaining prostheses that can improve the health, function, and quality of life of edentulous individuals. Continued interdisciplinary research and clinical validation will be essential to bring these visionary concepts into routine clinical practice.

### 4.2. Smart Dentures

The development of smart dentures represents a significant innovation in prosthodontics, transforming conventional prosthetic devices into interactive, multifunctional platforms that can monitor, diagnose, and even treat various oral and systemic conditions. These technologies aim to improve denture performance, enhance patient care, and facilitate remote clinical monitoring, particularly for the elderly and chronically ill.

Smart dentures are equipped with embedded sensors and microelectronic components that enable real-time monitoring of functional parameters in the oral cavity. For example, sensors capable of detecting occlusal forces, chewing cycles, and jaw movements have been integrated into the denture base or occlusal surfaces [38]. A recent study presents a new methodology for accurately measuring jaw kinematics: the use of low-profile electromagnetic field sensors suitable for clinical and research environments. The study combined these measurements with occlusal anatomy derived from intraoral scans and finite element model simulations to perform three-dimensional (3D) dynamic occlusal load calculations during biting and chewing [38].

In addition to functional monitoring, smart dentures can serve as intraoral monitoring tools for oral hygiene and disease prevention. Researchers have developed dentures with embedded temperature and pH sensors to detect early signs of infection, inflammation, or microbial colonization. According to Sultan et al. (2020), these devices can continuously assess the intraoral environment and alert patients to clean their dentures or seek professional care [39]. Some experimental models even incorporate biosensors that can measure salivary biomarkers such as glucose, lactate, or urea, potentially aiding in the early detection and monitoring of systemic diseases such as diabetes and renal dysfunction [40].

Beyond diagnostics, smart dentures have also been explored as drug-delivery systems. Recent advances in stimuli-responsive materials and hydrogel matrices have enabled the controlled release of therapeutic agents from the denture base in response to environmental cues. These theranostic prostheses can deliver antifungal, anti-inflammatory, or antimicrobial compounds in cases of denture stomatitis or mucosal irritation. One study demonstrated the feasibility of incorporating nanocarriers into oral appliances that respond to changes in pH or temperature, facilitating targeted and on-demand treatment without systemic side effects [41].

Despite their potential, the widespread clinical use of smart dentures is not without challenges. Significant improvements in denture material and techniques have made denture fabrication more patient-friendly, with a shorter production time and fewer inherent errors. However, the process can still be challenging for some patients. Distortion of wax and casts from this process can lead to a poor-fitting framework, pressure-induced mucosal lesions, and ridge resorption. A recently published in vitro study showed that RPD frameworks fabricated by SLM printing resulted in a better fit than traditional lost-wax and metal casting techniques. Another author reported that 3D-printed frameworks provide more uniform pressure, reducing the risk of ridge resorption [42].

As the population ages and the demand for high-quality, patient-centered prosthetic care increases, smart dentures may become a standard component of digital dentistry. By merging prosthodontics with bioengineering, materials science, and information technology, this innovative approach has the potential to dramatically improve oral health care and support the transition to precision, preventive, and connected dental medicine.

### 4.3. Artificial Intelligence and Digital Workflow

Artificial intelligence (AI) is increasingly influencing the field of prosthodontics, and its integration into the digital workflow for complete denture therapy represents a major advancement in precision dentistry. Traditionally, full-denture fabrication has relied heavily on the clinical judgment and manual skills of the practitioner. However, with the advent of AI technologies—particularly machine learning and data-driven design—complete dentures can now be manufactured with greater standardization, accuracy, and personalization.

One of the most significant applications of AI in this area is the automation of the denture-design process. Using datasets derived from thousands of successful denture cases, AI algorithms can analyze patient-specific anatomical features and suggest optimal tooth arrangements, occlusal schemes, and base contours [43]. These systems reduce the need for subjective decision-making and can dramatically reduce the time required for design and verification [44]. Mitsias et al. (2021) highlighted that AI-based tools can simulate natural occlusal dynamics and anatomical esthetics by referencing digital libraries and facial scans, leading to functionally and esthetically superior results [44].

Furthermore, AI can be embedded in diagnostic imaging and intraoral scanning systems to improve data acquisition and interpretation. Algorithms trained to identify patterns in oral structures, soft-tissue mobility or bone morphology can improve the accuracy of digital impressions and jaw relation records [45]. This enables more accurate simulations using virtual articulators and better prediction of denture performance in functional conditions. When coupled with 3D facial scanning, AI can also support smile design and esthetic integration, enhancing customization and patient satisfaction [45,46].

In the laboratory, AI-driven quality-control systems are being used to inspect milled or printed denture bases for dimensional accuracy, internal defects or surface irregularities. These systems can reduce manufacturing errors, optimize material usage, and standardize product quality across multiple manufacturing sites. Such automation also facilitates scalability and consistency in large clinical or commercial dental laboratories [44,46].

AI makes a significant contribution to the predictive aspect of prosthetic care. By analyzing longitudinal data from patients, including oral anatomy, previous prosthetic outcomes, and behavioral factors, machine learning models can predict the likelihood of denture-related complications such as sore spots, fractures, or dissatisfaction. This allows clinicians to proactively adjust designs or treatment plans prior to fabrication. Moreover, integration with electronic health records can enable AI systems to provide decision support, offering evidence-based recommendations for materials, retention systems, or the need for implant support [43,45,46].

Despite these advances, the use of AI in workflows for complete dentures also raises important considerations. The quality of the data used to train AI models is critical; biased or incomplete datasets can lead to errors or inappropriate generalizations. In addition, the ethical management of patient data, especially in cloud-based or interconnected systems, requires strict adherence to privacy and cybersecurity standards. Continued research and clinical validation are necessary to ensure that AI-enhanced workflows not only increase efficiency but also uphold the core principles of patient-centered care and safety [44,45,46].

As digital dentistry continues to evolve, the integration of artificial intelligence promises to redefine the restorative workflow. It offers clinicians powerful tools to streamline design, improve treatment outcomes, and enhance both clinical and patient experience. With further development, AI is likely to become an integral part of routine denture fabrication, enabling the development of a new standard of precision prosthodontics.

### 4.4. Sustainability in Denture Fabrication

As environmental concerns gain global prominence, dentistry—particularly prosthodontics—is being challenged to reevaluate its material consumption, waste generation, and overall environmental footprint. Traditionally associated with single-use materials, chemical processing and energy-intensive methods, fabrication of full dentures is undergoing a necessary transformation toward sustainability and environmentally responsible practices. This shift aligns with the broader healthcare mandate to reduce the system’s contribution to global emissions and material waste without compromising clinical efficacy [47].

Traditional denture manufacturing involves the use of polymethylmethacrylate (PMMA) resins, plaster, alginates, silicone impression materials, and petroleum-based polymers, most of which are not recyclable and contribute to clinical and laboratory waste. In addition, traditional workflows generate significant amounts of gypsum waste, chemical byproducts and disposable acrylic materials, especially during try-ins, adjustments, and remakes. These contribute not only to material costs, but also to landfill burden and occupational exposure to volatile organic compounds (VOCs) and monomer fumes [47,48].

The introduction of digital dentistry has created an opportunity to significantly reduce waste and energy consumption. Milled restorations made from prepolymerized PMMA blocks eliminate the need for flasks, investments, and multiple processing chemicals. Compared to conventional methods, subtractive CAD–CAM production results in more consistent quality and fewer defects, reducing the need for re-makes [6,49]. Furthermore, 3D-printing technologies, while still evolving in terms of mechanical properties, are more material-efficient and generate less environmental waste due to their additive nature. These digital workflows also require fewer physical appointments, indirectly reducing the carbon footprint associated with patient travel [6,50].

Another emerging area of sustainability is the use of biodegradable and recyclable materials. Researchers are exploring new generations of denture-base resins synthesized from plant-based monomers, such as polylactic acid (PLA), which offer reduced toxicity and improved environmental degradation. While current formulations may not yet meet all clinical durability standards, advances in polymer science are expected to bring bio-based materials closer to clinical adoption [25,51].

In addition to materials and manufacturing, the concept of green dental laboratories and environmentally conscious clinical protocols is gaining attention. Practices such as digital record-keeping (reducing paper waste), water-efficient equipment, and chemical-waste-management protocols are part of this larger sustainability framework. Educating clinicians and dental technicians about green alternatives and promoting a culture of resource conservation can have a cumulative positive impact on the sustainability of dental practices [48].

The initial investment required for digital infrastructure can be a barrier, especially for smaller practices. The environmental impact of e-waste and the energy consumption associated with CAD–CAM machines and 3D printers must also be considered. Therefore, life-cycle analyses of digital versus conventional denture-fabrication methods are essential to understand the true environmental costs of technological innovation [6,25].

In summary, integrating sustainability into total prosthodontics requires a multidimensional approach that includes material innovation, digital transformation and responsible clinical behavior. As environmental responsibility becomes an essential aspect of health care, prosthodontics must continue to adapt by embracing innovations that align oral health care with environmental stewardship.

## 5. Challenges and Considerations

Although digital and regenerative innovations promise substantial benefits, their equitable adoption requires reductions in cost barriers, practitioner training, and rigorous long-term clinical validation. Despite their potential, smart dentures raise concerns regarding cost, patient compliance, data privacy, and the durability of embedded technologies in the oral environment [49,50].

While the landscape of total-denture therapy is rapidly evolving through technological and material advances, several challenges remain that must be addressed to ensure equitable, safe, and effective clinical implementation. These include clinical limitations, economic barriers, ethical concerns, and the need for continuing professional education [51,52].

One of the most persistent challenges is the accessibility and affordability of advanced prosthetic care. Despite the increasing availability of digital workflows, implant-retained options, and high-performance materials, these modalities remain financially prohibitive for a significant portion of the edentulous population, especially in low-income settings and among older individuals with limited insurance coverage [49,51]. As a result, conventional acrylic-based full dentures remain the most feasible solution for many, underscoring the need for improvements in traditional techniques alongside digital innovations.

From a clinical perspective, complete dentures are still limited in their ability to replicate natural oral function. Even with optimal fabrication, low arch stability, mucosal discomfort, and adaptation issues are commonly reported, particularly in patients with highly resorbed ridges or poor neuromuscular coordination [20]. Although implant-based solutions mitigate these concerns, they are not suitable for all patients for reasons such as anatomical limitations, comorbidities, or patient refusal of surgical procedures.

The adoption of digital technologies and artificial intelligence also presents a learning curve for dental practitioners and technicians. Competence in digital impression systems, CAD software, and 3D-printing technologies is not yet universal, and training programs must continually adapt to equip clinicians with the necessary skills. Without proper training, the risk of suboptimal outcomes and misuse of digital tools may undermine their potential benefits [19].

Furthermore, as smart prostheses and AI-powered workflows become more prevalent, concerns about data security, patient privacy, and the ethical use of technology must be addressed. The collection, transmission, and storage of intraoral sensor data or facial scans raise critical questions about compliance with privacy regulations and the transparency of AI decision-making algorithms [45]. Ensuring informed consent, secure data handling, and algorithmic accountability will be central to maintaining trust in digital prosthetics.

From a regulatory perspective, the approval and standardization of new materials and devices—especially smart and regenerative prostheses—present additional hurdles. Many innovative materials are still in the preclinical or investigational phase and require robust evidence of safety, biocompatibility, and long-term performance before they can enter mainstream practice. This is particularly true for biomimetic liners, hydrogel-based drug-delivery dentures, and sensor-integrated devices, which must undergo thorough validation under real-world clinical conditions [37].

Finally, although sustainability is increasingly recognized as a priority, it still competes with the desire for quick, low-cost solutions. Achieving an environmentally conscious dental practice requires not only technological solutions, but also a cultural shift in professional practice toward long-term thinking and ethical stewardship of resources [47].

## 6. Conclusions

Complete denture therapy remains a foundational element of oral rehabilitation, particularly in aging and underserved populations. This review underscores the ways in which technological and scientific innovations are redefining the landscape of denture fabrication, function, and clinical care.

### 6.1. Key Findings

Digital innovations, including CAD–CAM and intraoral scanning, have been shown to enhance precision, patient comfort, and prosthesis durability. Material advancements, including nanofillers, high-impact PMMA, and bioactive liners, have been shown to enhance mechanical and biological performance. The integration of smart prosthetics with embedded sensors and responsive drug-delivery systems holds considerable promise for the real-time diagnostics and personalized care that such technology facilitates. The utilization of artificial intelligence facilitates the automation of design processes, the implementation of predictive modelling methodologies, and the execution of quality-control measures for complete dentures. Efforts to promote sustainability have led to a reduction in material waste and an increase in the use of biodegradable and recyclable prosthetic components.

### 6.2. Implications for Practice and Policy

The integration of digital and AI tools necessitates updated clinician training and revised educational curricula. The development of ethical standards for data management and AI transparency is imperative. Greater support is needed to expand access to advanced prosthetic care in resource-limited settings.

### 6.3. Future Directions

The study of this subject will entail the conduction of long-term clinical trials, the objective of which will be to assess the efficacy and safety of smart and regenerative prosthetics. The utilization of standardized validation protocols for artificial intelligence (AI) systems employed within the domain of prosthodontics will be imperative. The implementation of life-cycle assessments will be instrumental in guiding the selection of sustainable materials and reducing the carbon footprint.

Advancements in complete denture therapy are progressing towards intelligent, biologically integrated, and sustainable systems. The distribution of these benefits, in terms of both accessibility and equity, will be instrumental in shaping the future of prosthodontic care.

## Figures and Tables

**Table 1:** Dentures—evolution from traditional to future perspectives.

Aspect	Traditional Dentures	Current Advances	Future Perspectives
Materials	Heat-polymerized PMMA	High-impact PMMA, flexible resins, antimicrobial additives	Biodegradable, bioactive, tissue-integrating materials
Fabrication Method	Manual processing, flasking	CAD–CAM milling, limited 3D printing	Full 3D bioprinting, AI-driven automated design
Support Mechanism	Mucosal support	Implant-supported overdentures (2–4 implants)	Tissue-integrated smart prosthetics
Impression Technique	Conventional (alginate, silicone)	Digital intraoral scanning (with analog backup)	Fully digital dynamic scanning with real-time modeling
Patient Experience Focus	Basic function, esthetics	Quality of life (QoL), comfort, speech, esthetics	Personalized, data-driven care with predictive outcomes
Maintenance	Frequent relines, hygiene issues	Better hygiene with antimicrobial bases	Self-monitoring dentures with embedded biosensors
Cost and Accessibility	Affordable but basic	Higher cost, improved results	Aim to democratize advanced care globally
Sustainability	High material waste	More efficient CAD–CAM use	Green materials and low-carbon fabrication methods

## Data Availability

Data sharing is not applicable to this article.
